# Novel chemiluminescent immunoassay to measure plasma aldosterone and plasma active renin concentrations for the diagnosis of primary aldosteronism

**DOI:** 10.1038/s41371-020-00465-5

**Published:** 2021-02-09

**Authors:** Kyoko Teruyama, Mitsuhide Naruse, Mika Tsuiki, Hiroki Kobayashi

**Affiliations:** 1 Marketing Department, Fujirebio Inc, Tokyo, Japan; 2 Endocrine Center, Ijinkai Takeda General Hospital, Kyoto, Japan; 3 Division of Endocrinology and Metabolism, NHO Kyoto Medical Center, Kyoto, Japan; 4 Division of Nephrology, Hypertension, and Endocrinology, Nihon University School of Medicine, Tokyo, Japan; 5 Section on Genetics and Epidemiology, Research Division, Joslin Diabetes Center, Boston, MA USA

**Keywords:** Adrenal gland diseases, Aldosterone

## Abstract

Determination of plasma aldosterone concentrations (PAC) and plasma active renin concentrations (ARC) is essential for the diagnosis of primary aldosteronism (PA). In Japan, although PAC and ARC are measured by radioimmunoassay and immunoradiometric assay, respectively, non-radioisotopic methods with better detection sensitivity, measurement accuracy, and technical simplicity are needed. We developed two-site sandwich chemiluminescent enzyme immunoassays (CLEIAs) to measure both PAC and ARC using monoclonal antibodies immobilized onto ferrite particles. The results of both assays are obtained simultaneously from a single plasma sample within 30 min using a fully automated system. The novel CLEIAs were validated using plasma samples from patients with PA (*n* = 52) and essential hypertension (*n* = 23). The PAC determined by the CLEIA was significantly correlated with that measured by liquid chromatography/mass spectrometry or conventional radioimmunoassay. The ARC determined by the CLEIA was significantly correlated with that measured by immunoradiometric assay. The limits of detection of the CLEIAs for PAC and ARC were 0.1 ng/dl and 0.04 pg/ml, respectively, which were better than those of conventional methods (PAC: 2.5 ng/dl; ARC: 5 pg/ml). The PAC and PAC/ARC ratio (ARR) were significantly higher, and the ARC significantly lower, in patients with PA than in those with essential hypertension. An ARR cut-off of 1.31 ng/dl per pg/ml showed a sensitivity of 96.2% and specificity of 78.3% for PA screening. The newly developed CLEIAs for measuring PAC and ARC could provide a clinically powerful alternative to conventional methods used for hypertension screening in clinical practice.

## Introduction

Primary aldosteronism (PA) is a major cause of secondary hypertension [1–5]. Considering the high prevalences of cerebrovascular and cardiovascular complications in patients with PA compared with patients with essential hypertension (EH) [5–7], early diagnosis of and specific treatments for hyperaldosteronism are essential. Determination of the plasma aldosterone concentration (PAC) and plasma renin activity (PRA) is indispensable for diagnosing PA, including screening, confirmatory testing, and subtype diagnosis by adrenal venous sampling according to clinical practice guidelines [8–10]. Since measurement of these markers by liquid chromatography/tandem mass spectrometry (LC-MS/MS) [11] is not indicated in Japan because of the national health insurance system [12] designating the cost of each laboratory investigation, radioimmunoassay (RIA) has been used to determine the PAC, ARC, and PRA in clinical practice. Various issues with RIAs, such as the usage and disposal of radioisotopic materials, complexity of the manual assay, poor traceability of certified reference materials, and low detection sensitivity at lower concentrations [13, 14], remain to be resolved.

We developed new CLEIAs for detecting PAC and plasma ARC characterized by high sensitivity, sufficient traceability back to the certified reference materials, and versatile and efficient implementation by an automated system.

## Subjects and methods

### Patients

Patients referred to Kyoto Medical Center for further investigation of hypertension were evaluated. Diagnostic criteria for diagnosing PA is shown in Supplementary. In short, PA was diagnosed if the screening test was positive and at least one confirmatory test showed positive results. EH was diagnosed if any secondary cause of hypertension was excluded. The numbers of patients with PA, EH, and other miscellaneous diseases were 52, 23, and 33, respectively. Other miscellaneous diseases include renovascular hypertension, non-functioning adenoma, pheochromocytoma, paraganglioma, ACTH-independent macronodular adrenal hyperplasia, subclinical Cushing syndrome, and adrenocortical carcinoma were used for method comparison using peripheral blood. Apart from peripheral blood, we also evaluated the level of PAC and ARC by RIA and CLEIA using 50 samples from 5 patients for adrenal vein blood, and 18 samples from 18 patients for urine, respectively.

As part of routine clinical investigations, blood samples were collected into EDTA-2Na tubes from the antecubital veins of patients in the sitting position for at least 30 min in the clinic. Plasma samples were stored at less than −20 °C and subjected to our CLEIA immediately after thawing to room temperature in a 20 °C water bath. In addition, plasma samples collected from the adrenal veins of patients with PA (*n* = 50) and urine samples collected from patients with hypertension (*n* = 18) were analyzed.

The study was conducted according to the tenets of the Declaration of Helsinki. All patients provided written informed consent. The study was approved by the ethical committee of Kyoto Medical Center (#17-105).

### Development of new CLEIAs

Our CLEIAs for measuring PAC and ARC were developed using two monoclonal antibodies against different epitopes of aldosterone (Fujirebio, Inc., Tokyo, Japan) and renin (Fujirebio, Inc.), respectively. The limit of detection (LoD), accuracy, precision, linearity, and recovery were determined for each CLEIA according to the recommendations of the Clinical and Laboratory Standards Institute (EP17-A2, EP05-A3, EP06-A). In addition, using the dedicated fully automated systems (LUMIPULSE^®^ Presto II and LUMIPULSE^®^ L2400) with CLEIAs enables simultaneous determination of PAC and ARC, respectively, from a single plasma sample, requiring ~20 min.

### CLEIA for PAC

To detect PAC, we developed a two-site sandwich immunoassay using two specific monoclonal antibodies (primary antibody: anti-aldosterone mouse monoclonal antibody; secondary antibody: anti-metatype chicken antibody recognizing the aldosterone–aldosterone monoclonal antibody complex) against different epitopes of aldosterone. The plasma sample (30 µl) was incubated with 250 µl reagent A containing the primary antibody immobilized onto ferrite particles and incubated for 10 min at 37 °C. After washing with magnetic beads, the sample was incubated with 250 µl reagent B containing the ALP-conjugated secondary antibody and incubated for 10 min at 37 °C. After washing the magnetic beads, the complex consisting of the primary antibody, ALP-conjugated secondary antibody, and ferrite particles was dispersed, and 200 µl solution containing 3-(2′-spiroadamantane)-4-methoxy-4-(3″-phosphoryloxy) phenyl-1, 2-dioxetane disodium salt (AMPPD) was added to measure chemiluminescence [15]. The urine aldosterone concentration was determined following the same methods after deconjugation of glucuronic acid by HCl and neutralization. The CLEIA for PAC was calibrated in-house using a certified reference material for aldosterone [16]. (Fig. S1). Interference with assays measuring ascorbic acid, hemoglobin, bilirubin, bilirubin-conjugate, chyle, and rheumatoid factor, as well as cross-reactivity with corticosterone, cortisol, dexamethasone, spironolactone, progesterone, and 18-hydroxycorticosterone were analyzed. The accuracy, precision, linearity, and recovery of the CLEIA were measured.

### Novel CLEIA for ARC

For ARC detection, we developed two-site sandwich immunoassay using two specific mouse monoclonal antibodies against different epitopes of renin. An anti-active renin mouse monoclonal antibody that binds to the active site of active renin specifically was used as the detection antibody. An anti-renin monoclonal antibody that recognizes the non-active site of renin was used as the capture antibody. The plasma sample (40 µl) was incubated with 250 µl reagent A containing the detection antibody immobilized to ferrite particles and incubated for 10 min at 37 °C. After washing with magnetic beads, the sample was incubated with 250 µl reagent B containing the ALP-conjugated capture antibody and incubated for 10 min at 37 °C. After washing the magnetic beads, the complex consisting of the detection antibody, ALP-conjugated capture antibody, and ferrite particles was dispersed, and 200 µl solution containing AMPPD was added to measure chemiluminescence. The CLEIA for ARC was calibrated using in-house human recombinant renin (human activated renin [GenBank accession number NM_000537], amino acids 67–406 with C-terminal HIS tag) as the reference material. The accuracy, precision, linearity, and recovery of the CLEIA were measured.

### Correlations between the novel CLEIA and conventional assays

Correlation and linear regression analyses were conducted to compare PAC measurements by our novel CLEIA with those by LC-MS/MS and a conventional RIA (SPAC-S Aldosterone kits, Fujirebio Inc.), and to compare plasma ARC measurements by our novel CLEIA with those by a conventional immunoradiometric assay (IRMA) (Renin IRMA FR, Fujirebio Inc.) and PRA by a conventional RIA (Renin activity FR, Fujirebio Inc.), respectively.

### Clinical validation of the novel CLEIAs

PAC, ARC, and the PAC/ARC ratio (ARR) determined by the CLEIAs were compared between patients with PA and patients with EH.

### Statistical analysis

PAC, ARC, and ARR were compared between the patient groups using the Mann–Whitney *U* test. In the method comparison study, regression analysis was performed using the Passing–Bablok method and Pearson’s correlation coefficients. Bland–Altman analysis was used to evaluate mean differences. The statistical significance was set at *P* ≤ 0.05. Statistical analyses were performed using Analyse-it software (Analyse-it, Ltd.) and SAS for Windows (version 9.4; SAS institute, Cary, NC).

## Results

### CLEIA for PAC

Three different concentrations of certified reference materials for ensuring the traceability of aldosterone (NMIJ CRM 6402-a) were assayed in duplicate using our CLEIA for PAC. The certainty of the CLEIA for measuring the certified reference materials ranged from 99 to 104% (Table 1).

**Table 1:** Traceability of the CLEIA against the certified reference materials of aldosterone with three different concentrations.

	Certified value (ng/dL)	Aldosterone concentration by the CLEIA (ng/dL)	Certainty of the CLEIA assay
CRM Level1	20.1	20.99	104%
CRM Level2	41.1	40.67	99%
CRM Level3	79.2	80.49	102%

The value of the certified reference material of aldosterone (NMIJ CRM 6402-a) was confirmed. The recovery ratio by CLEIA against its certified value were 99–104%.

The LoD of the CLEIA for PAC was 0.1 ng/dl, and the upper limit of the measurement range was 200 ng/dl. There was no significant cross-reactivity of the CLEIA with corticosterone, cortisol, dexamethasone, spironolactone, progesterone, or 18-hydroxycorticosterone. There was no significant interference with the assays measuring ascorbic acid, hemoglobin, bilirubin, bilirubin-conjugate, chyle, or rheumatoid factor. The accuracy was 94–98% (Table S1a), and the coefficient of variation, as an indicator of the precision, was 1.7–3.0% (Table S2a). The dilution linearity of the samples with concentrations differing from the expected value was 96–104% (Table S3a). The recovery rate was 97–101% (Table S4a).

**Fig. 1:**
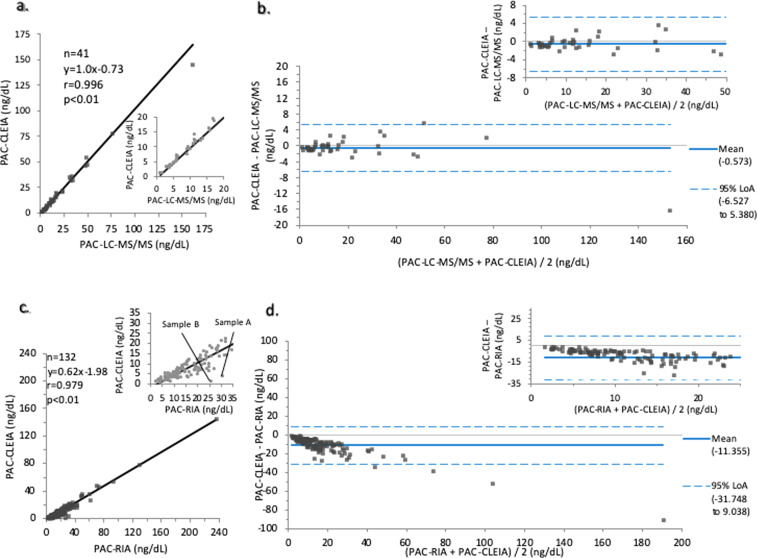
Correlations of the plasma aldosterone concentration (PAC) in peripheral blood measured by chemiluminescent enzyme immunoassay (CLEIA). Correlations between CLEIA and liquid chromatography/ mass spectrometry (LC-MS/MS) (**a**). Bland–Altman analysis was performed to analyze the correlations of the CLEIA results with those of LC-MS/MS (**b**). Correlations of the plasma aldosterone concentration (PAC) in peripheral blood measured by chemiluminescent enzyme immunoassay (CLEIA) with that measured by radioimmunoassay (RIA) (**c**). The PAC in samples A and B showed significant discrepancy between the CLEIA and RIA measurements and was measured by LC-MS/MS as well. Bland–Altman analysis was performed to analyze the correlations of the CLEIA results with those of RIA (**d**). The correlation between the three methods was investigated by Passing–Bablok regression analysis.

The PAC in the peripheral blood measured by the CLEIA was significantly correlated with the PAC measured by LC-MS/MS (*y* = 1.0x − 0.73, *r* = 0.996, *p* < 0.01; *n* = 41) (Fig. 1a). There was a significant correlation between the results of the two assay methods even for PACs < 10 ng/dl. The mean difference was −0.573 (95% Limits of agreement [LoA]: −6.53 to 5.38) (Fig. 1b). Although the PAC measured by the CLEIA showed a significant correlation with that measured by conventional RIA (*y* = 0.62x − 1.98, *r* = 0.979, *p* < 0.01; *n* = 132) (Fig. 1c), the correlation between the CLEIA and conventional RIA results was weaker than that between the CLEIA and LC-MS/MS results. The correlation was especially weaker when the PAC was <10 ng/dl (*y* = 0.39x − 0.19, *r* = 0.634, *p* < 0.01; *n* = 86). To validate the CLEIA measurements, the PAC of two discrepant samples A and B in Fig. 1c, was determined by LC-MS/MS. The LC-MS/MS result was similar to the CLEIA result (Table　2), with a mean difference of −11.4 (95% Limits of agreement [LoA]: −31.8 to 9.04) (Fig. 1d).

**Table 2:** Comparison of PAC between three assay methods in two samples with dissociated results between CLEIA and RIA.

Sample no.	CLEIA (ng/dL)	RIA (ng/dL)	LC-MS/MS (ng/dL)
Sample A	3.36	30.8	4.0
Sample B	0.76	26.0	1.17

Sample A and B showed discrepant results between CLEIA and RIA in Fig. 1c. These samples were measured with LC-MS/MS and compared to the value of RIA and CLEIA in Table 2. CLEIA and LC-MS/MS showed similar values.

**Fig. 2:**
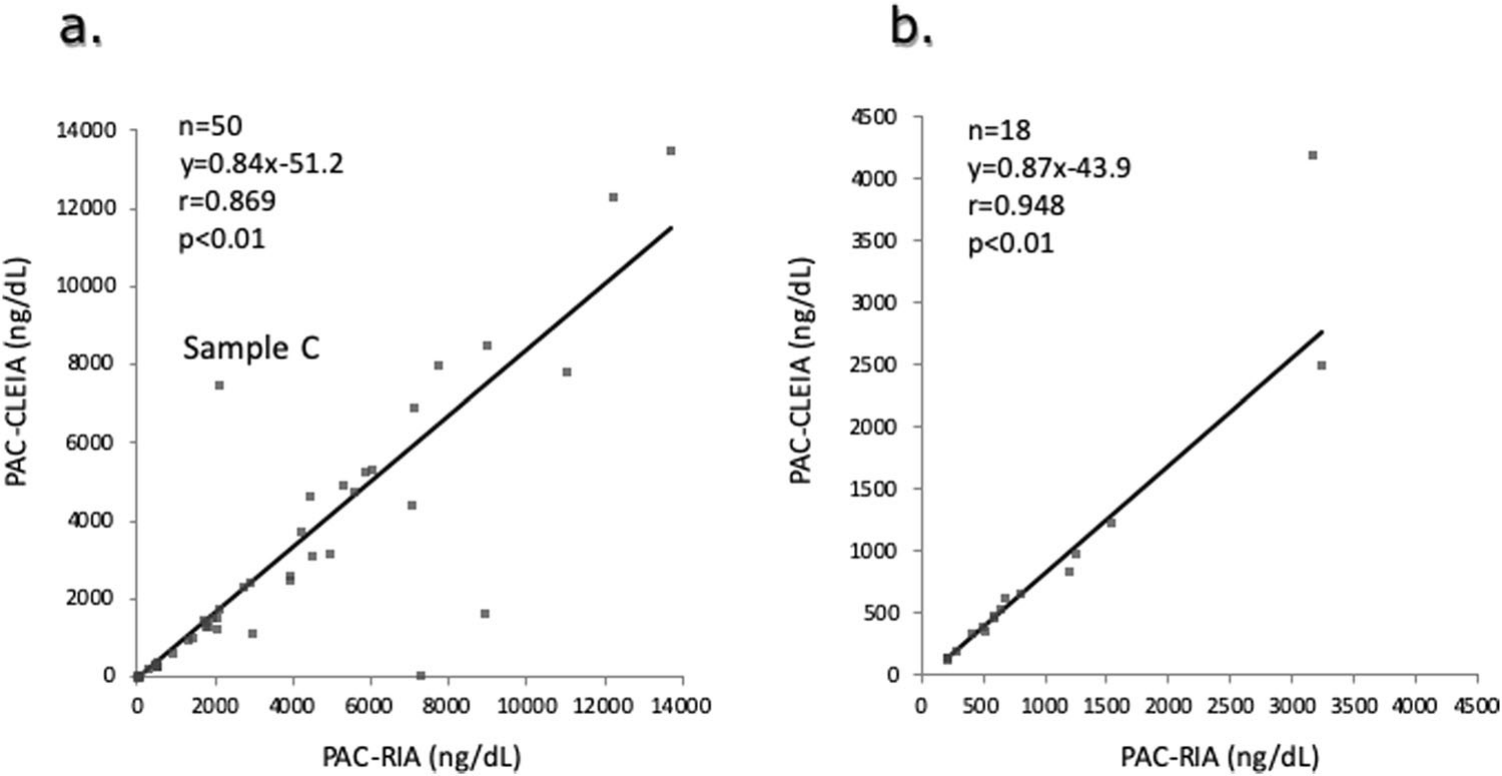
Conrrelations of the plasma aldosterone concentration (PAC) in adrenal vein blood or urine. Correlations of the PAC in adrenal vein blood (**a**) or the urine aldosterone concentration (**b**) between CLEIA and RIA. The correlation between the two methods was investigated by Passing–Bablok regression analysis.

PAC in the adrenal vein blood measured by the CLEIA was significantly correlated with that measured by conventional RIA. Although PAC in the adrenal vein blood measured by the CLEIA was significantly correlated with the PAC measured by RIA, the obtained values were higher in the RIA than CLEIA (*y* = 0.84x − 51.2, *r* = 0.869, *p* < 0.01; *n* = 50) (Fig. 2a). The PAC of sample C in Fig. 2a, which showed a significant difference between the CLEIA and RIA (7453.3 vs. 2120 ng/dl, respectively), measured 7462.9 ng/dl by LC-MS/MS. The urine aldosterone concentration measured by the CLEIA was correlated significantly with that measured by RIA (Fig. 2b).

### CLEIA for ARC

**Fig. 3:**
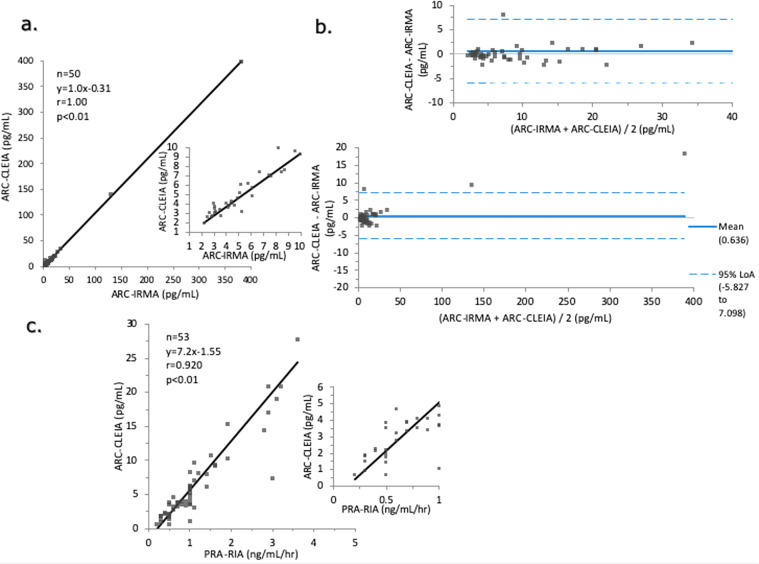
Correlation of the plasma active renin concentration (ARC) and plasma renin activity (PRA). Correlation of the ARC in the peripheral blood between chemiluminescent enzyme immunoassay (CLEIA) and immunoradiometric assay (IRMA) (**a**). Bland–Altman analysis was performed to analyze the correlation between the ARC measured by the CLEIA and that measured by IRMA (**b**). Correlations of the plasma ARC and PRA in the peripheral blood between the CLEIA and radioimmunoassay (RIA) (**c**). The correlation between the two methods was investigated by Passing–Bablok regression.

The LoD of the CLEIA for ARC was 0.04 pg/ml, and the upper limit of the measurement range was 1000.00 pg/ml, respectively. The accuracy of the CLEIA was 100–106% (Table S1b), and the coefficient of variation, as an indicator of the precision, was 3.0–4.1% (Table S2b). The dilution linearity of the samples with concentrations differing from the expected value was 97–104% (Table S3b). The recovery rate was 93–97% (Table S4b). The ARC measured by the CLEIA was significantly correlated with that measured by IRMA (*y* = 1.0x − 0.31, *r* = 1.00, *p* < 0.01; *n* = 50) (Fig. 3a). The mean difference was 0.636 (95% Limits of agreement [LoA]: −5.83 to 7.10) (Fig. 3b). The ARC measured by the CLEIA was significantly correlated with the PRA measured by RIA (*y* = 7.2x − 1.55, *r* = 0.920, *p* < 0.01; *n* = 53) (Fig. 3c).

### Clinical validation of the CLEIAs for PAC and ARC

**Fig. 4:**
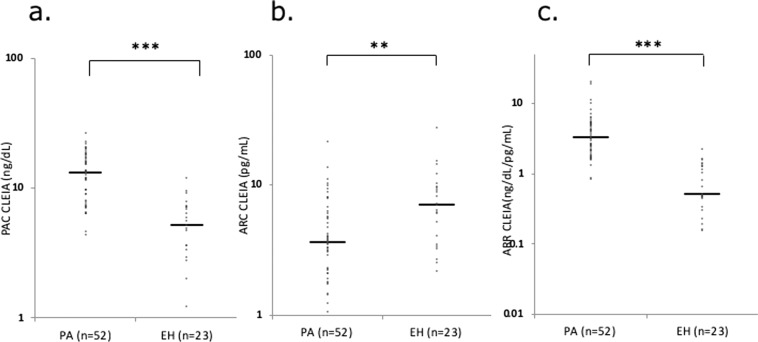
The plasma aldosterone concentration (PAC) (**a**), plasma active renin concentration (ARC) (**b**), and PAC/ARC ratio (ARR) (**c**) in patients with primary aldosteronism (PA) and those with essential hypertension (EH). ***p < 0.001, **p < 0.01, *p < 0.05.

The CLEIA PAC and plasma ARC measurements were compared between the patients with PA and those with EH. The baseline clinical characteristics of the patients are shown in Table 3. The PAC was significantly higher, and the plasma ARC was significantly lower, in patients with PA (PAC: 13.1 ng/dl; ARC: 3.7 pg/ml) than in patients with EH (PAC: 5.2 ng/dl; ARC: 7.1 pg/ml) (Fig. 4a, b). The plasma ARC measured by the CLEIA in all patients with PA above 0.2 pg/ml, which was higher than the 0.2 pg/ml analytic sensitivity of the CLEIA.

**Table 3:** Baseline characteristics of patients with PA and EH.

	PA (*n* = 52)	EH (*n* = 23)
Age (years)	61 (52–68)	59 (49–73)
Sex, males/females	24/51	11/17
Body mass index (kg/m^2^)	22.7 (21.2–26.3)	25.3 (23.6-–28.7)
Systolic blood pressure (mmHg)	129 (122–144)	133 (125–145)
Diastolic blood pressure (mmHg)	79 (71–90)	76 (71–90)
eGFR (mL/min/1.73 m^2^)	62.8 (54.0–73.9)	69.4 (62.4–84.5)
Baseline PAC (ng/dL)	13.2 (7.98–17.3)	5.24 (3.02–7.24)
Baseline PRA (ng/mL/h)	0.8 (0.4–1.0) (*n* = 24)	1.1 (1.0–1.9) (*n* = 13)
Baseline ARC (pg/mL)	3.70 (2.10–5.92)	7.05 (3.61–9.69)

Continuous data are shown as the median (interquartile range).

*PA* primary aldosteronism, *EH* essential hypertension, *eGFR* estimated glomerular filtration rate, *PAC* plasma aldosterone concentration, *PRA* plasma renin activity, *ARC* active renin concentration.

**Fig. 5:**
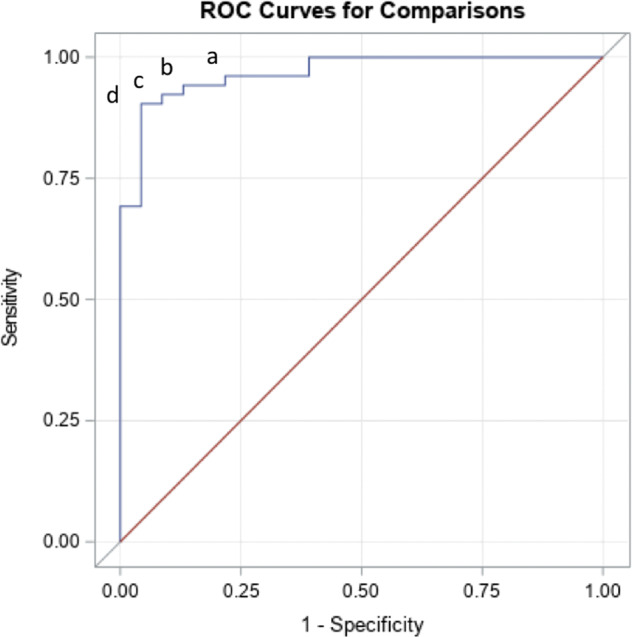
Receiver operating characteristic analysis of the plasma aldosterone concentration (PAC)/active renin concentration (ARC) ratio (ARR) measured by the chemiluminescent enzyme immunoassay (CLEIA) for screening primary aldosteronism (PA). An ARR cutoff of 1.31 ng/dl per pg/ml had the best sensitivity (96.2%) for diagnosing PA, with a specificity of 78.3% (area under the curve [AUC]: 0.967; 95% CI: 0.932–1.000) (**a**). An ARR cutoff of 1.51 ng/dl per pg/ml had a sensitivity of 94.2% and specificity of 87.0% (**b**). An ARR cutoff of 1.62 ng/dl per pg/ml had a sensitivity of 92.3% and specificity of 91.3% (**c**). An ARR cutoff of 1.65 ng/dl per pg/ml had a sensitivity of 90.4% and specificity of 95.7% (**d**).

The ARR measured by the CLEIA was significantly higher in patients with PA (3.31 ng/dl per pg/ml) than in patients with EH (0.52 ng/dl per pg/ml) (Fig. 4c). Receiver operating characteristic (ROC) analysis was performed to investigate the diagnostic value of the ARR for distinguishing PA from EH. The optimal ARR cutoff for discriminating PA from EH was 1.31 ng/dl per pg/ml, which yielded an area under the ROC curve of 0.967 (95% CI: 0.932–1.000), sensitivity of 96.2%, and specificity of 78.3% (Fig. 5).

In this study, the conversion formulas between conventional and the new assays were as follows: PAC (CLEIA) = 0.62 × PAC (RIA) − 1.98, and PAC (CLEIA)/PRA (RIA) = − 1.05 + 0.49 × PAC (RIA)/PRA (RIA). For example, the ARR cut-off value of 20 determined by RIA for PAC and PRA, which is used to screen for PA, can be converted to 8.75 by PAC (CLEIA)/PRA (RIA). The PAC cut-off value of 6 determined by RIA, which is used to diagnose PA by the saline infusion test, can be converted to 1.74 by PAC (CLEIA).

## Discussion

The prevalence of PA has reached 5–10% in the hypertensive population [9, 17, 18]. Since patients with PA are more likely to experience cardiovascular and cerebrovascular diseases compared with patients with EH [19–22], an early and accurate diagnosis of PA is of great importance. However, there are methodological issues with RIAs for measuring PAC, ARC, and PRA, which are critical for the screening and diagnosis of PA. In this study, we developed novel CLEIAs for measuring PAC, urine aldosterone concentration, and plasma ARC. These CLEIAs demonstrated good traceability to the certified reference material of aldosterone, good linearity over a wide range of concentrations, and good correlation with the LC-MS/MS results both in plasma and urine samples.

These findings agree with previous reports of the advantages of CLEIAs [23–25]. Japan’s health and medical services include a universal health insurance system that covers the medical costs of all citizens. However, the cost of LC-MS/MS is not covered by national insurance. Hence, RIA is the only assay method for PAC covered by Japanese health insurance; yet, this assay has demonstrated limitations in sensitivity and reproducibility, especially at lower concentration ranges [13] as well as at higher concentration ranges in adrenal vein samples. In agreement with a previous study [13], our CLEIAs demonstrated small but significant differences from the RIA results, especially at concentrations <10 ng/dl. However, the CLEIA results were identical to the LC-MS/MS results, even at lower concentrations. Since the optimal cutoff values for confirmatory tests (saline infusion test and fludrocortisone suppression test) exceed 5.0–10 ng/dl according to the clinical practice guidelines set by the Endocrine Society [9], measurement of relatively low PACs is important in decision making of the confirmatory tests. For PAC measurements, our CLEIA showed a good correlation with LC-MS/MS and good sensitivity and accuracy at concentrations <10 ng/dl. Thus, it could be a useful tool for the diagnosis of PA.

Our CLEIA for measuring the plasma ARC demonstrated a good correlation with conventional assays. The CLEIA showed a detection sensitivity of 0.04 pg/ml, which was significantly better than that (5 pg/ml) of IRMA. The detection sensitivity of the ARC is important for determining the ARR, which is universally used for PA screening in patients with hypertension [8–10]. The conventional assay, with a relatively high LoD, has a higher risk of false-negative results in PA screening. The CLEIA, which showed an improved sensitivity, is expected to provide evidence for more detailed cut-off values for screening and accurate diagnosis of PA. Concerning the cut-off value of ARR, Morimoto et al [14]. used the Accuraseed ARC assay to demonstrate that an ARR cut-off of 6.0 ng/dl per pg/ml had ~100% sensitivity for diagnosing PA. By contrast, Manolopoulou et al [26]. used CLEIA to demonstrate that an ARR cut-off of 1.12 ng/dL per μIU/ml (equivalent to 1.87 ng/dL per pg/ml) had a sensitivity of 98.9% for diagnosing PA. In agreement with the study by Manolopoulou et al., our ROC analysis of the ARR determined by the novel CLEIA showed the best sensitivity (96.2%) at a cutoff of 1.31 ng/dl per pg/ml. One possible explanation for the discrepancy in cut-off values among studies is the varying degree of hyperaldosteronism among the different PA subtypes. The prevalence of aldosterone-producing adenoma with a higher PAC than that in idiopathic hyperaldosteronism was 60% in the study by Morimoto et al [14]. and 13% in the present study, respectively. Further study is needed to determine the optimal cut-off of ARR for screening PA with consideration of the different PA subtypes.

The present CLEIA is applicable as a standard assay method for measuring the PAC in daily clinical practice, and an automated system with these CLEIAs enables us to obtain PAC and plasma ARC results within 30 min. This significant decrease in measurement time will help improve the efficiency of the PA diagnostic process in the clinic and the success rate of adrenal venous sampling. The novel CLEIAs for PAC and ARC could affect the clinical practice of hypertension and PA.

### Limitations

The present study has several limitations. First, blood sampling in the patients was not optimized in terms of food intake, posture, time of day, or use of antihypertensive medications, which may affect PAC and/or ARC measurements. However, the major aim of the present study was to validate the new CLEIAs and compare their efficacies with those of conventional assays. The sampling conditions likely did not affect the correlations between the assays. Second, an ARR cut-off in the current study is calculated by small sample size. Further systematic studies using larger numbers of samples obtained under standardized conditions are needed to establish the appropriate cut-offs of PAC and ARR for screening PA.

### Summary table

#### What is known about the topic

- Radioimmunoassay (RIA) has been used to determine the PAC, ARC, and PRA in clinical practice, although various issues with RIAs, such as the usage and disposal of radioisotopic materials, complexity of the manual assay, poor traceability of certified reference materials, and low detection sensitivity at lower concentrations, remain to be resolved.

#### What this study adds

- The novel CLEIAs using a fully automated system developed to measure PAC and ARC were characterized by a better detection sensitivity, much shorter measurement time, and lower cost compared with the conventional RIA. These new methods are expected to facilitate the diagnostic process and improve the quality of hypertension and PA clinical practice.

## Supplementary information

Supple Figure 1

SUPPLEMENTAL METHODS
