# Supplementary figures and images for: Novel chemiluminescent immunoassay to measure plasma aldosterone and plasma active renin concentrations for the diagnosis of primary aldosteronism
Source: J Hum Hypertens. 2021 Feb 9;36(1):77–85. doi: 10.1038/s41371-020-00465-5 (PMC8766281; doi:10.1038/s41371-020-00465-5)

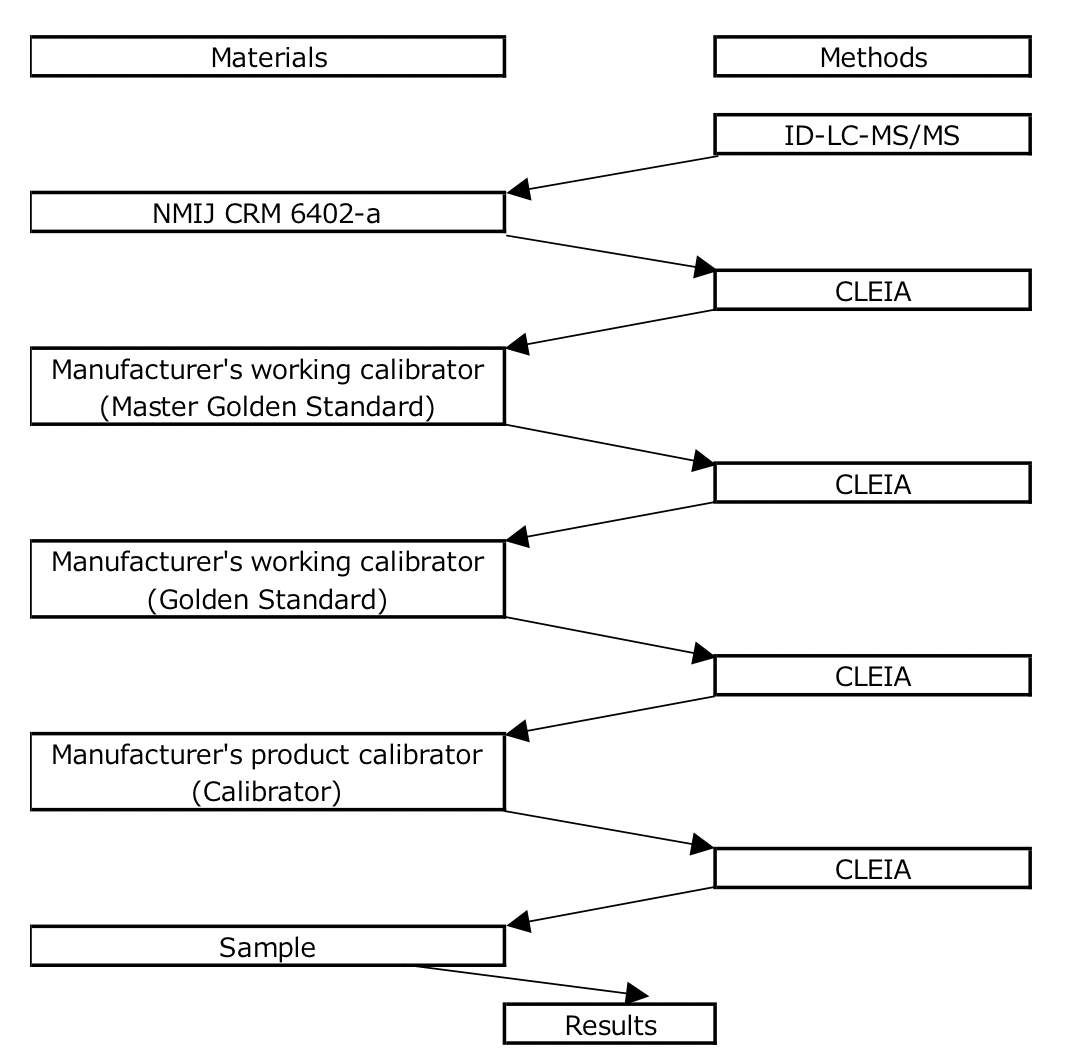

Supplement: Supplementary file 1 — Supple Figure 1 [file 41371_2020_465_MOESM1_ESM.png]
