# Supplementary material for: Novel chemiluminescent immunoassay to measure plasma aldosterone and plasma active renin concentrations for the diagnosis of primary aldosteronism
Source: J Hum Hypertens. 2021 Feb 9;36(1):77–85. doi: 10.1038/s41371-020-00465-5 (PMC8766281; doi:10.1038/s41371-020-00465-5)
Supplement: Supplementary file 2 — SUPPLEMENTAL METHODS [file 41371_2020_465_MOESM2_ESM.docx]

**SUPPLEMENTAL**

**Diagnosis of primary aldosteronism**

Primary aldosteronism (PA) was diagnosed based on the guidelines of the Japan Endocrine Society^1^ and the Japan Society of Hypertension.^2^ Hypertensive patients with an elevated plasma aldosterone/renin ratio (ARR, >20 with plasma aldosterone concentration [PAC] expressed in ng/dL and plasma renin activity [PRA] in ng/mL/h) underwent at least one confirmatory test (saline infusion, captopril challenge, and furosemide upright posture).

**REFERENCES**

1. Nishikawa T, Omura M, Satoh F, Shibata H, Takahashi K, Tamura N, Tanabe A; Task Force Committee on Primary Aldosteronism, The Japan Endocrine Society. Guidelines for the diagnosis and treatment of primary aldosteronism--the Japan Endocrine Society 2009. Endocr J 2011 **58** 711–721. (doi: 10.1507/endocrj.EJ11-133)
2. Shimamoto K, Ando K, Fujita T, Hasebe N, Higaki J, Horiuchi M, Imai Y, Imaizumi T, Ishimitsu T, Ito M, Ito S, Itoh H, Iwao H, Kai H, Kario K, Kashihara N, Kawano Y, Kim-Mitsuyama S, Kimura G, Kohara K, Komuro I, Kumagai H, Matsuura H, Miura K, Morishita R, Naruse M, Node K, Ohya Y, Rakugi H, Saito I, Saitoh S, Shimada K, Shimosawa T, Suzuki H, Tamura K, Tanahashi N, Tsuchihashi T, Uchiyama M, Ueda S, Umemura S; Japanese Society of Hypertension Committee for Guidelines for the Management of Hypertension. The Japanese Society of Hypertension guidelines for the management of hypertension (JSH 2014). Hypertens Res 2014 **37** 253–390. (doi: 10.1038/hr.2014.20)

Figure legend

Figure. S1

Diagram of ensuring traceability of aldosterone concentration by the new CLEIA assay using NMIJ CRM 6402-a as certified reference materials

Supplemental Table S1a. Accuracy in CLEIA of PAC.

|  | PAC (ng/dL) | Accuracy |
| --- | --- | --- |
| sample1 | 9.43 | 98% |
|  | 9.32 | 97% |
|  | 9.41 | 98% |
|  | 9.37 | 97% |
|  | 9.37 | 97% |
|  | 9.38 | 97% |
| sample2 | 75.45 | 97% |
|  | 75.34 | 97% |
|  | 75.36 | 97% |
|  | 75.34 | 97% |
|  | 75.71 | 98% |
|  | 74.79 | 97% |
| sample3 | 127.32 | 94% |
|  | 128.96 | 95% |
|  | 128.78 | 95% |
|  | 128.61 | 95% |
|  | 129.41 | 96% |
|  | 128.84 | 95% |

Plasma samples were taken 6-fold measurements.

Supplemental Table S1b. Accuracy of ARC in CLEIA

|  | ARC (pg/mL) | Accuracy |
| --- | --- | --- |
| sample1 | 21.25 | 106% |
|  | 21.16 | 105% |
|  | 21.12 | 105% |
|  | 21.01 | 105% |
|  | 20.99 | 104% |
|  | 21.03 | 105% |
| sample2 | 417.04 | 102% |
|  | 415.41 | 102% |
|  | 419.53 | 103% |
|  | 412.14 | 101% |
|  | 416.86 | 102% |
|  | 417.21 | 102% |
| sample3 | 712.28 | 101% |
|  | 707.49 | 100% |
|  | 716.30 | 102% |
|  | 708.69 | 101% |
|  | 717.96 | 102% |
|  | 705.62 | 100% |

Plasma samples were taken 6-fold measurements.

Supplemental Table S2a. Precision of PAC in CLEIA

| Sample | | n | Mean (ng/dL) | Within-run CV | Between-run CV | Between-day CV | Total CV |
| --- | --- | --- | --- | --- | --- | --- | --- |
| plasma | 1 | 24 | 9.76 | 1.0% | 1.3% | 0.0% | 1.7% |
|  | 2 | 24 | 75.32 | 1.3% | 1.5% | 1.7% | 2.6% |
|  | 3 | 24 | 135.59 | 2.4% | 0.9% | 1.5% | 3.0% |

A 6-day and 2-run per day precision test was performed.

Supplemental Table S2b. Precision of ARC in CLEIA.

| Sample | | n | Mean (pg/mL) | Within-run CV | Between-run CV | Between-day CV | Total CV |
| --- | --- | --- | --- | --- | --- | --- | --- |
| plasma | 1 | 28 | 20.42 | 0.7% | 0.9% | 3.1% | 3.3% |
|  | 2 | 28 | 239.85 | 0.9% | 0.6% | 2.8% | 3.0% |
|  | 3 | 28 | 441.66 | 1.1% | 0.7% | 3.8% | 4.1% |

A 7-day and 2-run per day precision test was performed.

Supplemental Table S3a. Dilution Linearity of PAC in CLEIA.

|  | | sample1 | Sample2 | Sample3 |
| --- | --- | --- | --- | --- |
| Value (ng/dL) | Undiluted* | 195.01 | 7462.90 | 2538.23 |
|  | 25-fold Dilution | 7.58 |  |  |
|  | 50-fold Dilution | 3.74 |  |  |
|  | 100-fold Dilution |  | 77.31 | 26.51 |
| Expected Value  (ng/dL) | 25-fold Dilution | 7.80 |  |  |
|  | 50-fold Dilution | 3.90 |  |  |
|  | 100-fold Dilution |  | 74.63 | 25.38 |
| Recovery Rate | 25-fold Dilution | 97% |  |  |
|  | 50-fold Dilution | 96% |  |  |
|  | 100-fold Dilution |  | 104% | 104% |

*Undiluted value of sample2 and sample3 are measured by LC-MS/MS because they are over the measurement range of CLEIA.

Supplemental Table S3b. Dilution Linearity of ARC in CLEIA.

|  | | sample1 | sample2 | Sample3 |
| --- | --- | --- | --- | --- |
| Value (pg/mL) | Undiluted | 1.83 | 7.03 | 24.80 |
|  | 2-fold Dilution | 0.93 | 3.67 | 12.80 |
|  | 5-fold Dilution | 0.36 | 1.46 | 5.06 |
|  | 10-fold Dilution |  | 0.71 | 2.52 |
|  | 100-fold Dilution |  |  | 0.25 |
| Expected Value  (pg/mL) | 2-fold Dilution | 0.92 | 3.52 | 12.40 |
|  | 5-fold Dilution | 0.37 | 1.41 | 4.96 |
|  | 10-fold Dilution |  | 0.70 | 2.48 |
|  | 100-fold Dilution |  |  | 0.25 |
| Recovery Rate | 2-fold Dilution | 101% | 104% | 103% |
|  | 5-fold Dilution | 97% | 104% | 102% |
|  | 10-fold Dilution |  | 101% | 102% |
|  | 100-fold Dilution |  |  | 100% |

Supplemental Table S4a. Recovery of PAC in CLEIA.

|  | Mean measured value of Control Sample (ng/dL) | Spiked amount (ng/dL) | Mean measured value of Test Sample (ng/dL) | recovery |
| --- | --- | --- | --- | --- |
| sample1 | 1.89 | 42.99 | 44.16 | 98% |
| sample2 | 4.60 | 109.41 | 110.47 | 97% |
| sample3 | 0.63 | 174.24 | 176.06 | 101% |

Test was performed by aldosterone solutions to base samples at a volume ratio of 1:9. The control samples were prepared similarly by adding specimen diluent to base samples.

Supplemental Table S4b. Recovery of ARC in CLEIA.

|  | Mean measured value of Control Sample (pg/mL) | Spiked amount (pg/mL) | Mean measured value of Test Sample (pg/mL) | recovery |
| --- | --- | --- | --- | --- |
| sample1 | 30.23 | 226.66 | 250.37 | 97% |
| sample2 | 20.73 | 606.99 | 605.84 | 96% |
| sample3 | 8.15 | 927.81 | 870.67 | 93% |

Test was performed by adding renin solutions to base samples at a volume ratio of 1:9. The control samples were prepared similarly by adding specimen diluent to base samples.
